# Time-Resolved Visual Chiral Discrimination of Cysteine Using Unmodified CdTe Quantum Dots

**DOI:** 10.1038/s41598-017-00983-2

**Published:** 2017-04-18

**Authors:** Forough Ghasemi, M. Reza Hormozi-Nezhad, Morteza Mahmoudi

**Affiliations:** 1 Department of Chemistry, Sharif University of Technology, Tehran, 11155-9516 Iran; 2 Institute for Nanoscience and Nanotechnology, Sharif University of Technology, Tehran, Iran; 3 Department of Nanotechnology and Nanotechnology Research Center, Faculty of Pharmacy, Tehran University of Medical Sciences, Tehran, 13169-43551 Iran; 4 Department of Anesthesiology, Brigham and Women’s Hospital, Harvard Medical School, Boston, Massachusetts 02115 United States

## Abstract

Herein, we demonstrate a simple yet novel luminescence assay for visual chiral discrimination of cysteine. Thioglycolic acid (TGA)-capped cadmium-telluride (CdTe) quantum dots (QDs) exposing green emission were directly synthesized in aqueous solution. The interaction between cysteine molecules and CdTe QDs induced the aggregation of QDs via hydrogen bonding. As a result of electronic coupling within these aggregates, a redshift both in the absorption and emission spectra of QDs occured. The difference in the kinetics of the interactions between L- and D-cysteine with CdTe QDs led to chiral recognition of these enantiomers. Addition of D-cysteine to CdTe QDs in a basic media caused a green-to-yellow color change, while no color alteration in QDs emission was observed in the presence of L-cysteine after 2 hours. Notably, the QDs used in the proposed assay are free from any labling/modification, which makes the present strategy highly attractive for sensing applications. Furthermore, the presented chiral assay is able to determine the enantiomeric excess (ee) of D-cysteine in the whole range of ee values (from −100% to 100%).

## Introduction

Chirality is known as a determinative feature in biological phenomena. Many drugs, bioactive compounds and organic molecules exist in chiral form. In general, two enantiomers of a chiral compound exhibit different biochemical activity, pharmaceutical and physiological effects, depending on their absolute configurations^1–4^. Enantiomeric recognition of amino acids, as essential bioactive substances and building blocks of proteins, polypeptides, and various drugs, is of vital importance in both process development and quality control^5–7^.

Various methods have been reported to distinguish the chirality of amino acids, including high performance liquid chromatography^8–10^, gas chromatography^11^, mass spectrometry^12^, electrochemistry^13–15^, and capillary electrophoresis^16, 17^. However, most of these techniques require complicated sample pretreatment and sophisticated instrumentation which makes them impractical for real-time analysis. Rapid enantiosensing technique, *i.e*., circular dichroism (CD), suffers from low sensitivity and low tolerance against contamination^18^. Thus, it is intriguing and useful to develop a rapid, simple, sensitive, and high-throughput assay especially, a solution-based sensor capable of visual discrimination of enantiomers. Colorimetric and fluorometric assays are highly demanded due to their ease of detection, even by naked eye and without the need of elaborate equipment.

Due to their unique optical properties, quantum dots (*e.g*., cadmium-telluride (CdTe) QDs)^19^, gold, and silver nanoparticles^20, 21^ possess a tremendous potential for applications in optical sensing. Reported colorimetric and fluorimetric chiral recognition probes for amino acids are based on either the inherent chirality of metal surfaces^22–24^ or chiral molecules adsorbed on the nanoparticle surface^7, 25–30^. Metallic nanoparticles with intrinsic chiral structure are numerable and the adsorption of chiral molecules onto the surface of nanoparticles leads to complex chiral detection.

**Figure 1:**
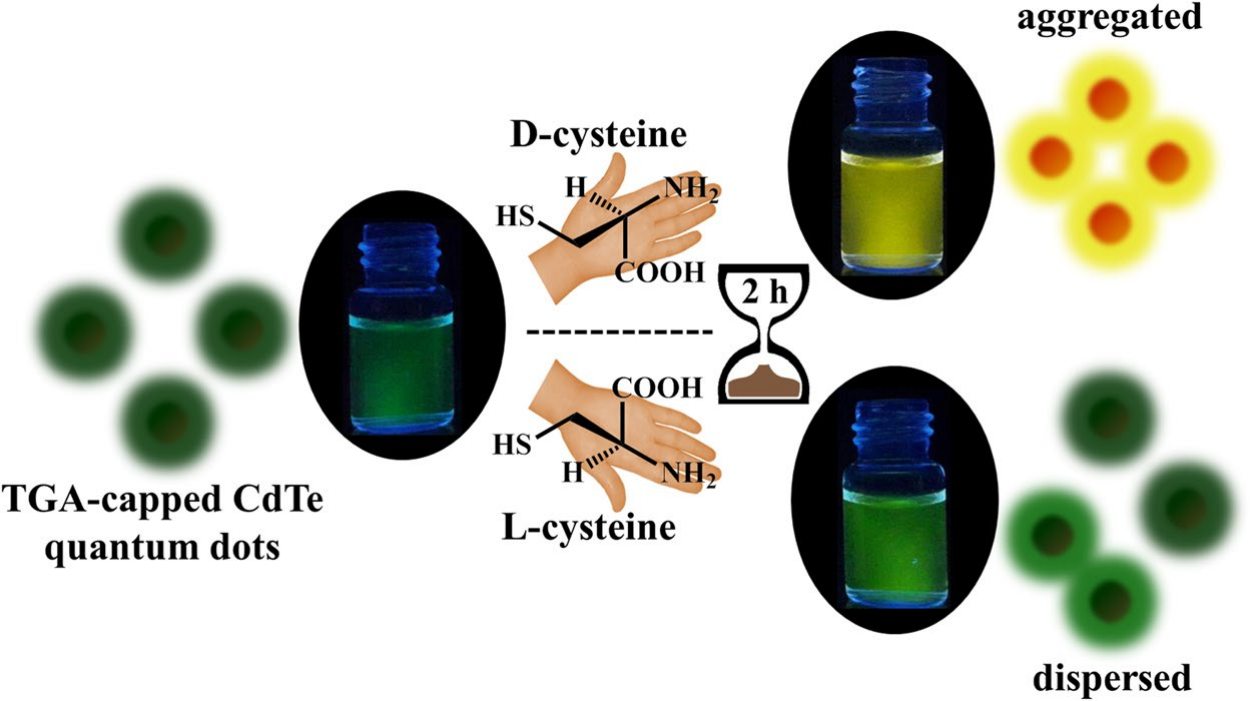
Schematic illustration of time-resolved visual chiral discrimination of cysteine using unmodified CdTe quantum dots.

In the present study we report a new strategy for chiral recognition in aqueous solution using CdTe QDs. We show that both D- and L-cysteine induce aggregation in CdTe QDs, but with different kinetics leading to distinct emission spectra and color change (see Fig. 1).

## Results and Discussion

### Time-resolved chiral recognition

**Figure 2:**
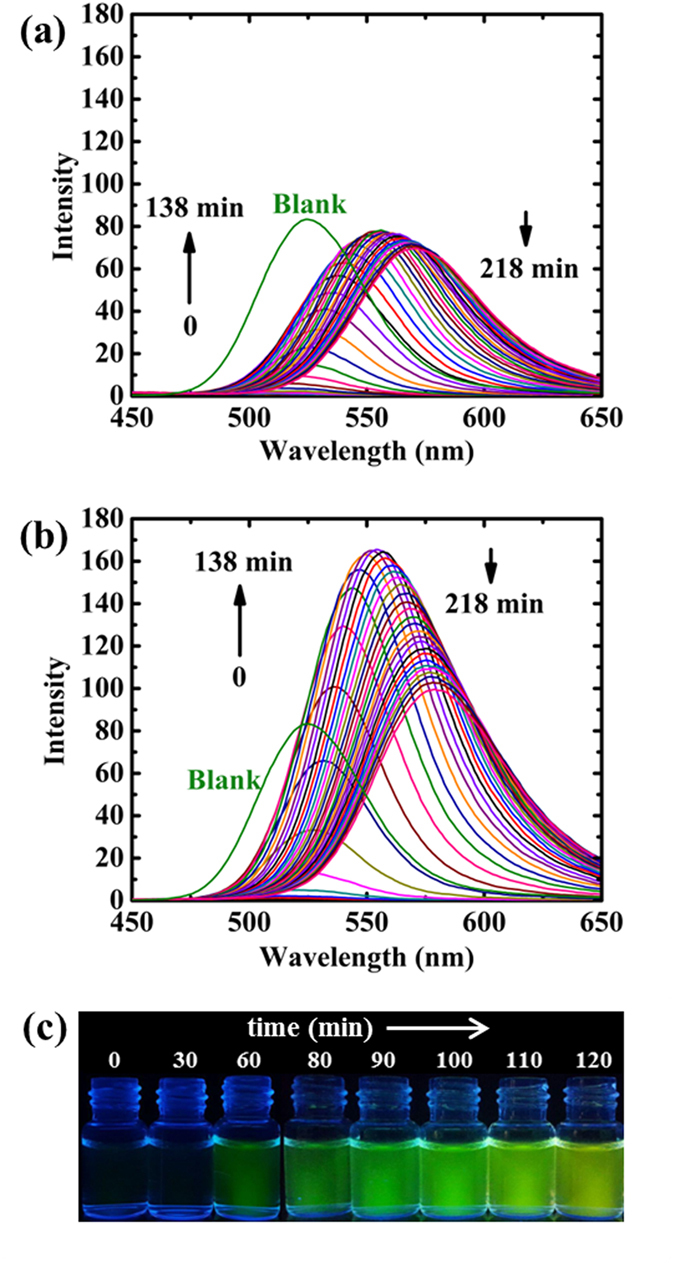
Time-course variation in emission spectra of CdTe QDs upon the addition of (**a**) L-cysteine and (**b**) D-cysteine (The concentrations of cysteine, NaOH, and QDs were 2 mmol L^−1^, 60 mmol L^−1^, and 4.6 µmol L^−1^, respectively; the excitation wavelength was 340 nm, time interval to record each spectrum was 6 min). (**c**) Fluorescence images of time-dependent luminescence of QDs after the addition of 2 mmol L^−1^ D-cysteine and 60 mmol L^−1^ NaOH under UV irradiation, the time of the images was from 0 min (left) to 120 min (right).

TGA-capped CdTe QDs exposing green emission were directly synthesized in aqueous solution. The absorption and fluorescence spectra of the as-prepared thioglycolic acid (TGA)-capped CdTe QDs are demonstrated in Fig. S1 in Supporting Information (SI). L- and D-cysteine were added to the QDs’ solution and after 15 min NaOH was inserted to the mixture. Time-course emission behavior of QDs toward D- and L-cysteine is shown in Fig. 2. The emission of QDs was quenched thoroughly upon the addition of cysteine in the basic media. Recovery in fluorescence-intensity and redshift of the spectral peak were thereafter observed. In the case of L-cysteine, the fluorescence intensity reached a maximum value after 138 min with an intensity very close to the emission of the blank (solution with no cysteine, shown in Fig. S2). However, for D-cysteine, it took 78 min to obtain the maximal fluorescence intensity which was twice as high as the blank. In both cases, after reaching a maximum, fluorescence quenching and further redshift in the emission peak were observed.

**Figure 3:**
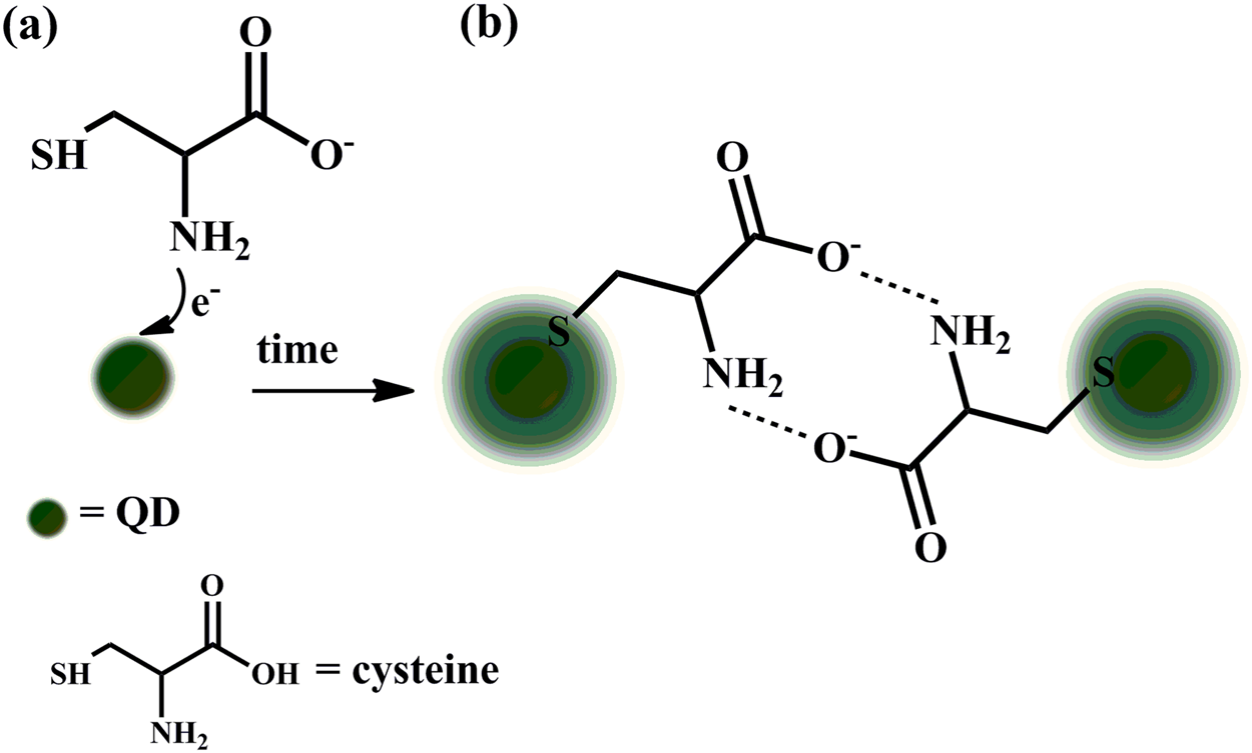
(**a**) Photoinduced electron transfer (PET)-mediated quenching of QD. (**b**) Fluorescence recovery of QDs by PET inhibition.

As shown in Fig. 2, the presence of cysteine in solution triggered fluorescence quenching which can be attributed to the photoinduced electron transfer (PET)^31^. Unprotonated amino groups are particularly suitable as electron donors in PET mechanism^32^. Therefore, in the presence of cysteine, containing an amino unit, PET can take place between the nitrogen lone pair and QDs, causing quenching (see Fig. 3).

Due to the high binding affinity of thiol groups toward Cd atoms at the surface of CdTe QDs, cysteine can attach to CdTe through the formation of Cd-S bonds^33, 34^. Therefore, after the addition of cysteine to QDs solution, cysteine was gradually bonded to the surface of QDs. It is assumed that the affinity of thiol to QDs can actually push the amino group away from the surface of QDs. Thus, considering the strong dependency of PET on the electron donor–fluorophore distance^32^, PET was suppressed and allowed the fluorescence to be switched on (see Fig. 3).

The rate of fluorescence-intensity recovery by D-cysteine was faster than by L-cysteine. In addition, a higher fluorescence-intensity was observed for D-cysteine (see Fig. 2). Compared to L-cysteine, D-cysteine exhibits a stronger interaction affinity to CdTe QDs^34^. Consequently, it is expected that a higher number of D-cysteine compounds can attach to the surface of QDs, which caused enhancement in both the rate of recovery and the emission (due to better passivation of the QDs’ surface)^35, 36^.

As demonstrated in Fig. 2, the redshift in the emission spectra was initiated upon the entrance of cysteine in the basic media. The presence of cysteine molecules on the surface of QDs induced the aggregation process due to the hydrogen bonding between the adsorbed thiols. Aggregation of QDs then caused a redshift in the emission spectra that can be explained by two possible reasons: (1) electronic coupling between neighbouring QDs in the aggregates which can lead to a redshift in both absorption and emission spectra; (2) exciton energy transfer between QDs in small distance which will not exhibit any redshift in the absorption spectra^37^. The observed redshift in the absorption spectra, cuased by the aggregation of QDs in the presence of cysteine (see Fig. S4), confirms that the redshift appeared in the emission spectra can be originated from electronic coupling between QDs.

Another point to be considered from Fig. 2 is that CdTe QDs displayed a gradual quenching after reaching a maximum emission intensity. In agreement with our findings, Koole *et al*.^37^ had reported that exciton energy transfer is absent in small green QDs, while it can occur in aggregated QDs. Furthermore, a reduction in fluorescence intensity can be induced by exciton energy transfer^37^. Over time, the size and the number of aggregates increases which leads to exciton energy transfer between QDs in one aggregate, and consequently causes emission quenching.

**Figure 4:**
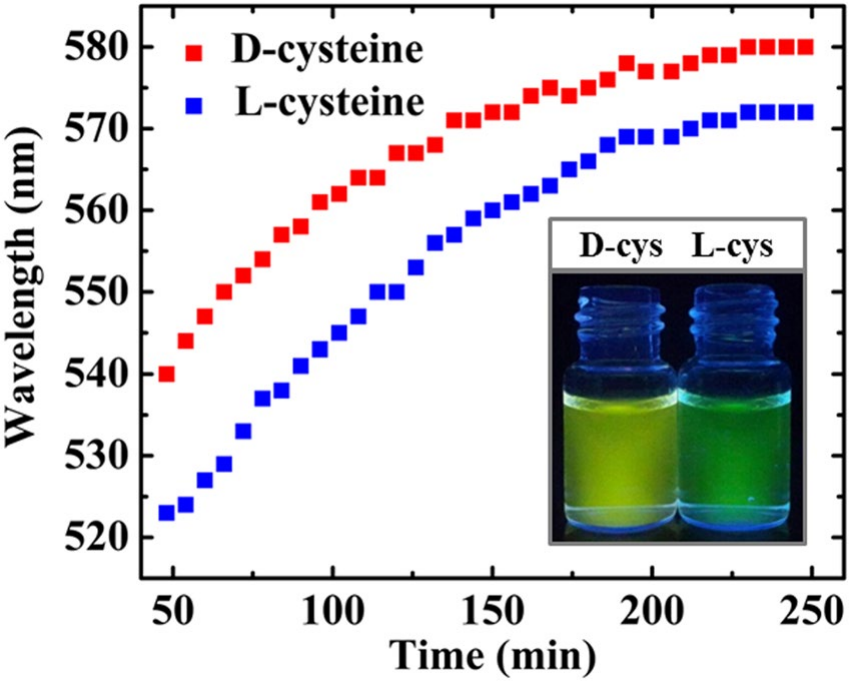
Changes in the wavelength of maximum fluorescence after the addition of D- and L-cysteine (2 mmol L^−1^) against time (min). The inset shows fluorescence images of QDs 2 h after the addition of 2 mmol L^−1^ L-, D-cysteine and 60 mmol L^−1^ NaOH under UV irradiation.

**Figure 5:**
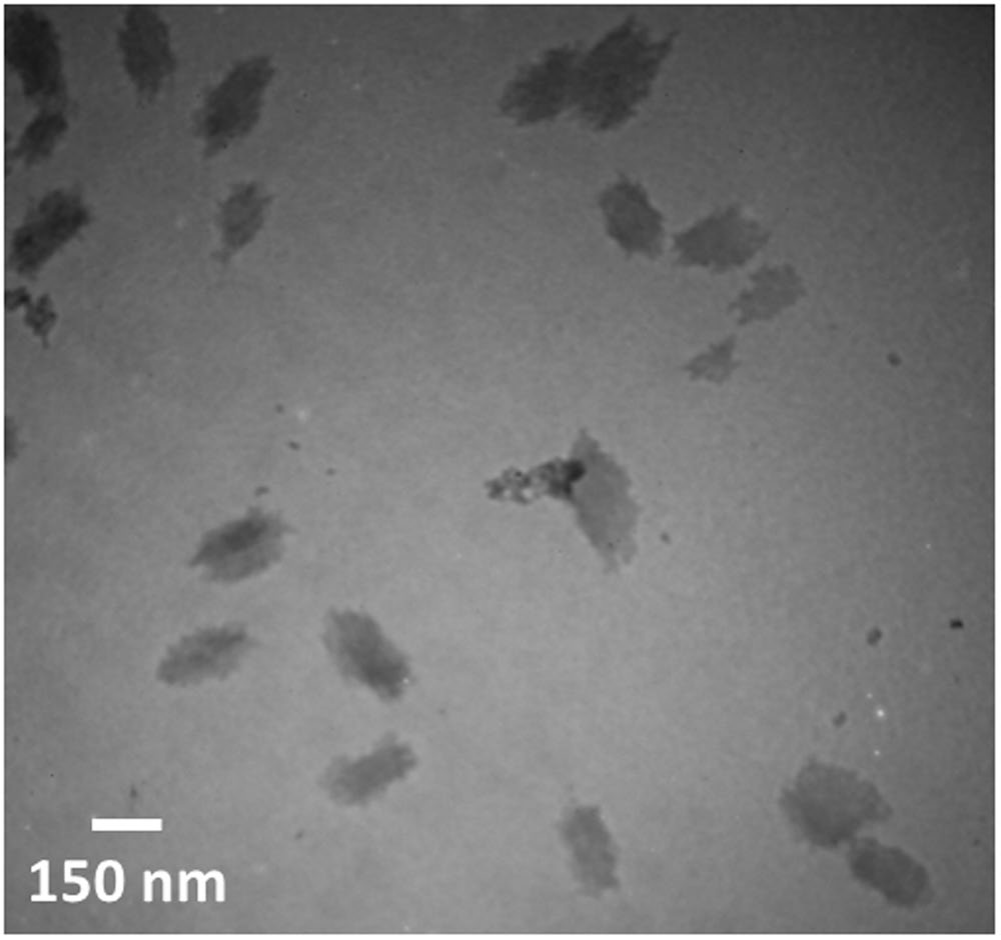
TEM images of CdTe QDs in the presence of D-cysteine.

Figure 4 illustrates the peak wavelengths related to the spectra given in Fig. 2, against time. In a certain time, the amount of redshift caused by D-cysteine was more than by L-cysteine. As mentioned earlier, the aggregation of QDs caused redshift in the emission spectra. Therefore, we hypothesized that the rate of aggregation is higher in the presence of D-cysteine, rather than L-cysteine. This can be used for visual discrimination between L- and D- forms of cysteine (see inset in Fig. 4). Over time, the difference in redshifts of QDs declined for both D- and L-cysteine. Dynamic light scattering (DLS) measurements confirm that D-cysteine mediates more aggregated QDs compared to L-cysteine (See Fig. S5). TEM images further confirmed the aggregation of QDs in the presence of cysteine (see Fig. 5). Figure 2c shows representative emitted colors from QDs over time after the addition of D-cysteine and NaOH under UV irradiation.

### Fluorometric determination of enantiomeric excess

**Figure 6:**
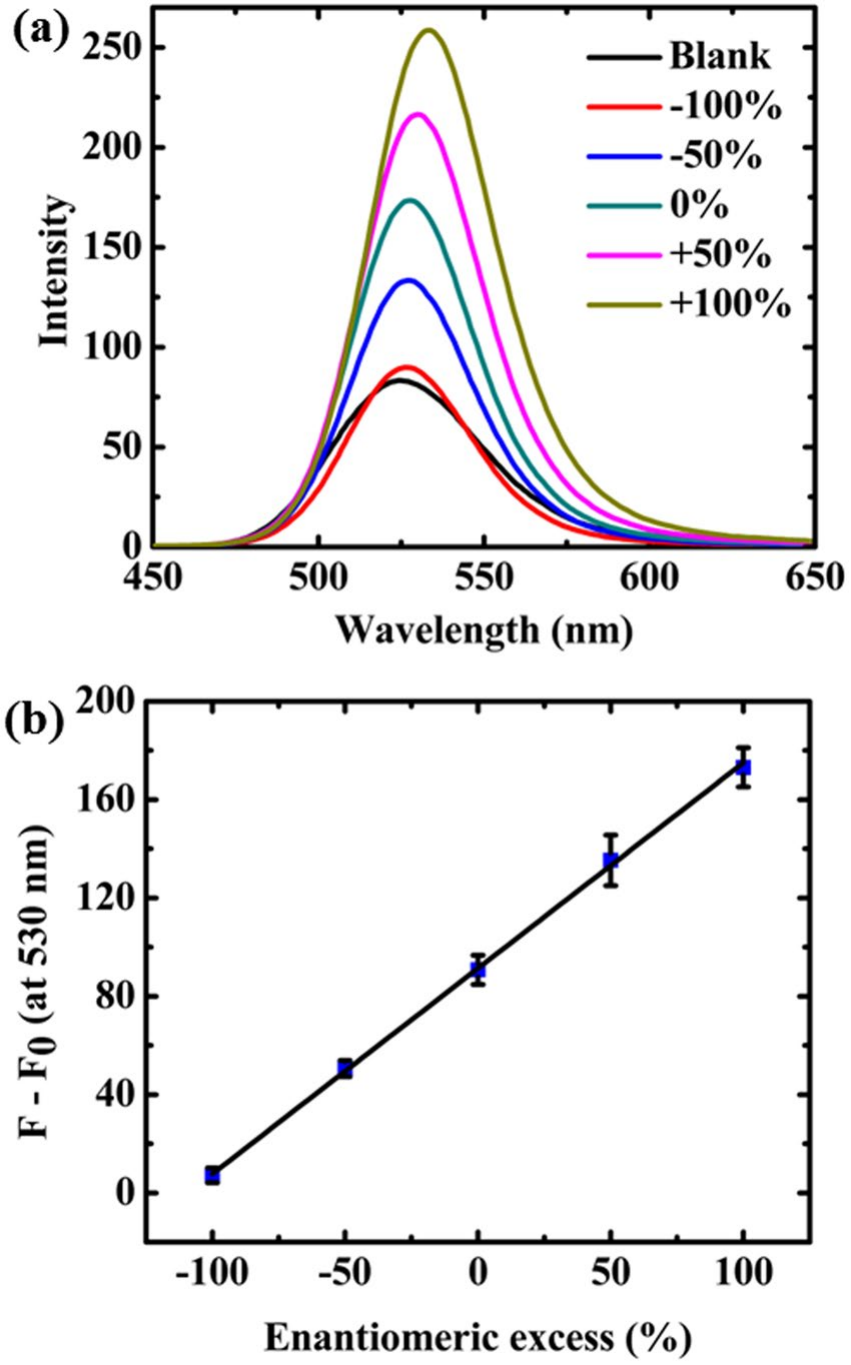
(**a**) Emission spectra of QDs in different enantiomeric excess of D-cysteine (the total concentration of cysteine was 1 mmol L^−1^). (**b**) The relationship between fluorescence intensity and enantiomeric excess of D-cysteine at total cysteine concentration of 1 mmol L^−1^.

Since it is essential to determine enantiomeric excess (*i.e*., the difference between the mole fractions of enantiomers) in chiral drug and asymmetric catalyst development^38, 39^, our chiral assay was applied to quantify the enantiomeric excess of cysteine. NaOH concentration, as an effective parameter on emission, was optimized. The difference between peak wavelengths of QDs’ emission in the presence of D- and L-cysteine (∆λ_D, L_) was monitored at different concentrations of NaOH (see Fig. S6). ∆λ_D, L_ rised up to 60 mmol L^−1^, and then no further change was observed. Therefore, we selected 60 mmol L^−1^ of NaOH as the optimal concentration for further experiments. The emission spectra of CdTe QDs in different enantiomeric excess of cysteine (total concentration 1 mmol L^−1^) are demonstrated in Fig. 6. The highest and lowest signals were observed for absolute D- and L-cysteine, respectively. A good linear relationship was obtained between emission signal at 530 nm and enantiomeric excess of D-cysteine.

### The response of unmodified CdTe QDs to other α-amino acids

**Figure 7:**
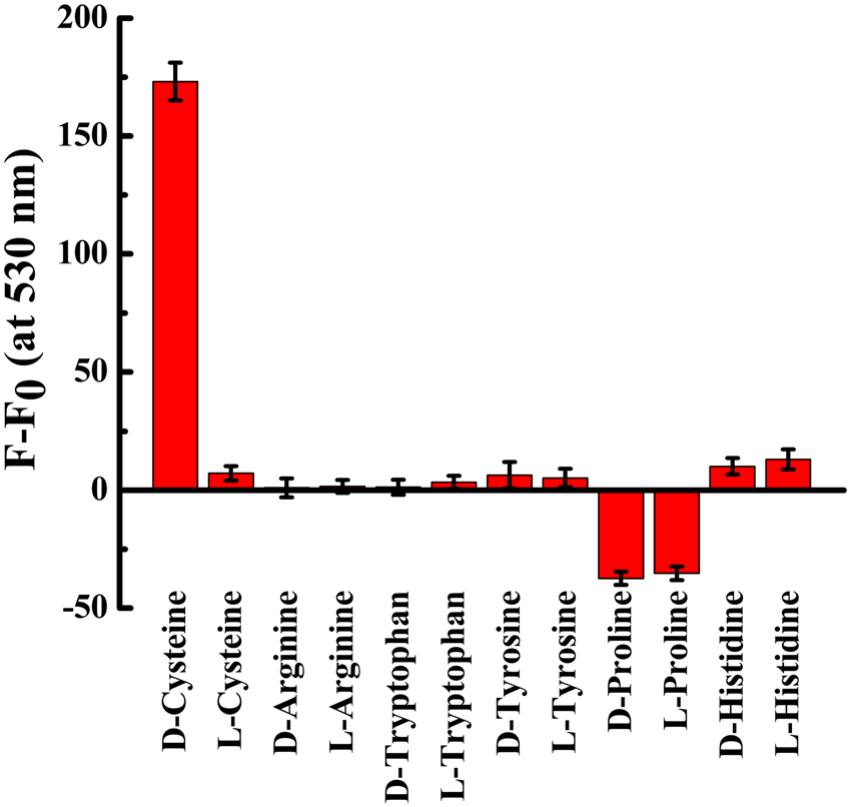
The responses of QDs to enantiomerically pure cysteine, arginine, tryptophan, tyrosine, proline and histidine amino acids at a concentration of 1 mmol L^−1^ (in the presence of 60 mmol L^−1^ NaOH).

The response of the unmodified CdTe QDs in the presence of other α-amino acids including arginine, tryptophan, tyrosine, proline, and histidine was negligible regrdless to their D- and L- forms (see Fig. 7). However, the response of D-cysteine was the highest and distinct from L-cysteine. All α-amino acids consist of a carboxylic acid and an amino functional group. However, cysteine is distinctive from the rest due to the presence of its thiol group. Considering that a chiral CdTe QD was employed in the proposed assay, the key role of the thiol group in the differentiation of L- and D-cysteine is evident. In fact, both D- and L-cysteine are bonded to the surface of QDs through S-Cd bond, but with a different strength that makes them distinguishable. The CD measurement (Fig. S7) confirmed that the applied CdTe QDs were achiral. Similarly, the CD spectra of L- and D-cysteine exhibited mirror-image profiles (see Fig. S8). Aditional CD features emerged for the mixture of QDs and cysteine, compared to pure cysteine (see Fig. S9). These features can be attributed to the formation of Cd_x_(cystine)_y_ complex and to the chirality induced by the absorbed cysteine molecules on QDs surface^34^. This assumption has been experimentally and theoretically proven by Zhou *et al*.^34^ studying CdTe QDs: since an atom with four different substituents produces a chiral center, if all four atomic positions in a tetrahedron formed by Te and Cd atoms are different, a chiral center will be formed. As they have presented, the adsorption of cysteine molecules on the surface of QDs induces four different substituents, resulting in a chiral center.

## Conclusions

In summary, a fluorometric chiral recognition assay was developed for detection of cysteine using unmodified CdTe QDs. We found that the rate of QDs’ aggregation was quite different in the presence of D- and L-cysteine. The chiral assay took advantage of the aggregation rate of unmodified QDs for enantioselective recognition of cysteines. The emission color variation of QDs revealed its promissing chiral detection of cysteine even by the naked eye. Moreover, this novel chiral sensor can be applied for the measurement of enantiomeric excess. Compared with other developed fluorimetric methods, the present strategy is fascinating mainly because the CdTe QDs employed do not require any labeling or modification with chiral molecules.

## Methods

### Reagents

Cysteine and other amino acids were purchased from Merck. Tellurium powder (Te), thioglycolic acid (TGA), Sodium borohydride (NaBH_4_), and cadmium chloride (CdCl_2_.2H_2_O) were purchased from Sigma. Deionized water was used throughout the experiment.

### Instrumentation

Absorbance spectra were recorded on a Lambda spectrophotometer from Perkin Elmer using 1.0 cm glass cell. The fluorescence spectra were measured on a Cary Eclipse fluorescence spectrometer (Varian) with the use of 1 × l cm quartz cell. All the spectra were recorded at room temperature. Size distributions were obtained using Zetasizer Viscotec 802 at ambient temperature. Circular dichroism (CD) measurements were made using Jasco J-810 CD recorder.

### Synthesis of TGA functionalized CdTe QDs

TGA-capped CdTe QDs were prepared according to the procedure described previously (Ghasemi *et al*. 2016^40^). Typically, 2.87 mmol of TGA was added to 10 mL of CdCl_2_ solution (0.29 mol L^−1^) and the pH was adjusted to 9.0 with NaOH solution. Then, the solution was stirred under argon bubbling for 5 min. 0.76 mmol of Te powder was separately mixed with 75 mL of NaBH_4_ solution (64 mmol L^−1^) under argon flow at 50 °C. After 20 min, 10 mL of pH-adjusted CdCl_2_ solution was added, and the temperature was increased to 150 °C. The resulting TGA-capped CdTe QDs with green emission (reaction time 45 min) were taken out and allowed to cool in ice bath.

## Electronic supplementary material

Supplementary information
