# Supplementary material for: Time-Resolved Visual Chiral Discrimination of Cysteine Using Unmodified CdTe Quantum Dots
Source: Sci Rep. 2017 Apr 18;7:890. doi: 10.1038/s41598-017-00983-2 (PMC5429850; doi:10.1038/s41598-017-00983-2)
Supplement: Supplementary file 1 — Supplementary information [file 41598_2017_983_MOESM1_ESM.doc]

**Time-Resolved Visual Chiral Discrimination of Cysteine Using Unmodified CdTe Quantum Dots**

**Forough Ghasemi^1^, M. Reza Hormozi-Nezhad^1^, ^2*^, Morteza Mahmoudi^3^, ^4*^**

^1^Department of Chemistry, Sharif University of Technology, Tehran, 11155-9516, Iran

^2^Institute for Nanoscience and Nanotechnology, Sharif University of Technology, Tehran, Iran

^3^Department of Nanotechnology and Nanotechnology Research Center, Faculty of Pharmacy, Tehran University of Medical Sciences, Tehran 13169-43551, Iran

^4^Department of Anesthesiology, Brigham and Women’s Hospital, Harvard Medical School, Boston, Massachusetts 02115, United States

*Corresponding authors: (M.R.H.-N.) email: [hormozi@sharif.edu](mailto:hormozi@sharif.edu);

(M.M.) email: [mmahmoudi@bwh.harvard.edu](mailto:mmahmoudi@bwh.harvard.edu)

**Content:**

1. The absorption and fluorescence spectra of the as-prepared TGA-capped CdTe QDs

2. Effect of NaOH on QDs’ emission

3. The result of dynamic light scattering (DLS) measurement for QDs

4. Red shift in absorption spectra of QDs in the presence of D- and L-cysteine

5. Intensity size distribution of QDs after the addition (a) L- and (b) D–cysteine

6. The effect of NaOH concentration on the difference between emission wavelengths of QDs in the presence of D- and L-cysteine

7. CD and absorption spectra of the as-prepared CdTe QDs

8. CD spectra of L- and D-cysteine

9. CD and absorption spectra of QDs in the presence of D- and L-cysteine

**1. The absorption and fluorescence spectra of the as-prepared TGA-capped CdTe QDs**

The maximum emission appears at 523 nm. The approximate size of the QDs is about 0.8 nm, as defined by the first absorption peak in the UV–visible spectra and the well-established empirical equation[^1^](#_ENREF_1). The result of DLS measurement for QDs, shown in Fig. S2, is in a good agreement with this value.


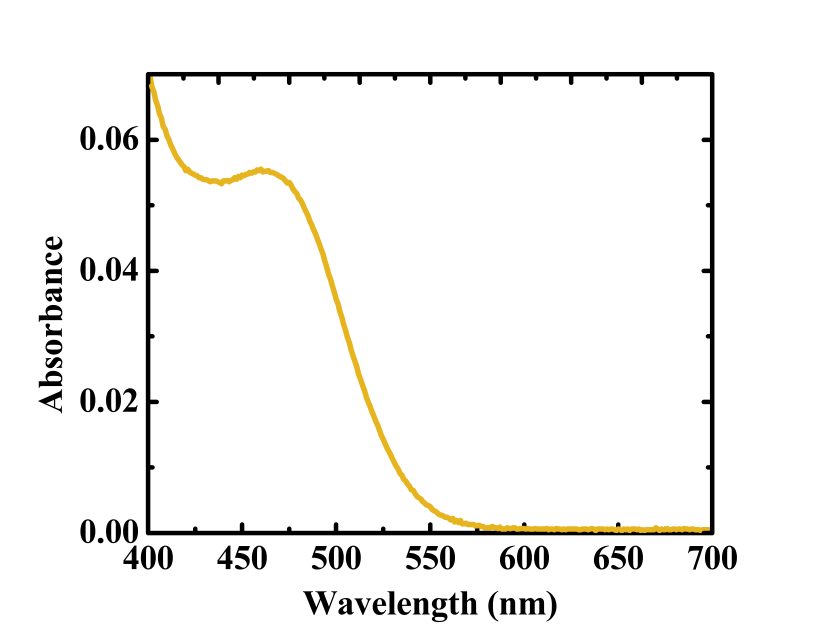


**(a)**


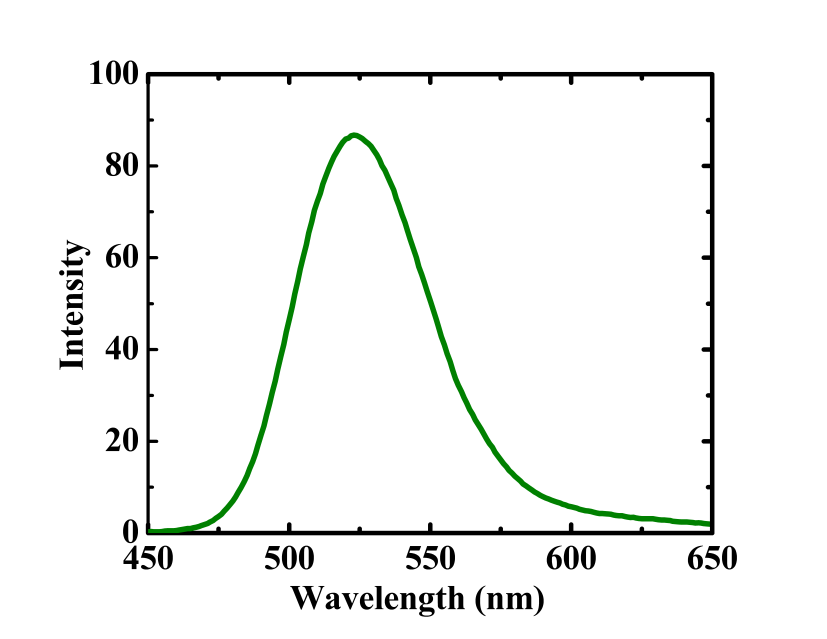

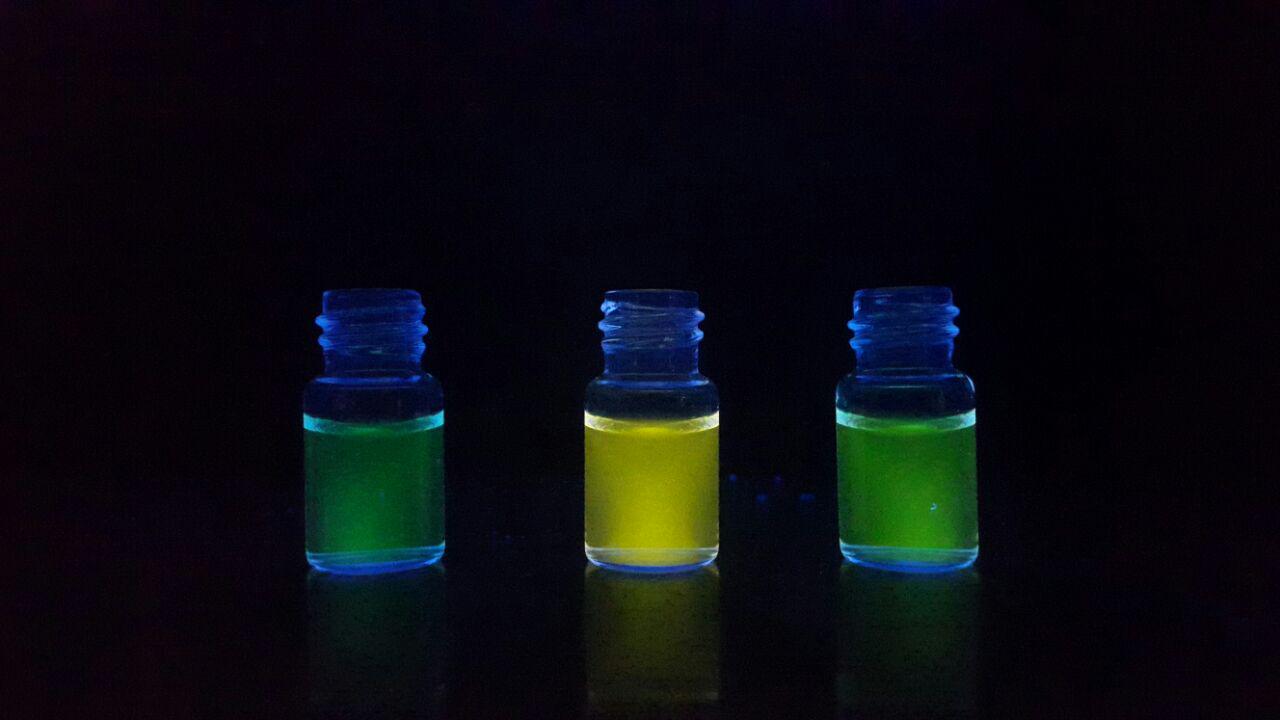


**(b)**


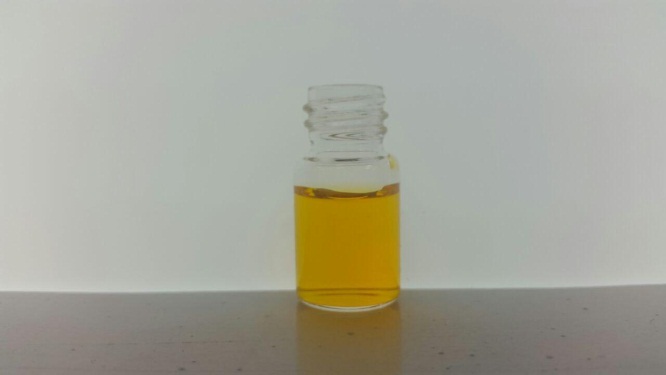


**Figure S1.** (a) The absorption spectrum of TGA-capped CdTe QDs. The inset shows a photograph of a solution containing the as-prepared QDs. (b) The emission spectrum of TGA-capped CdTe QDs (excited at 340 nm). The inset shows a corresponding fluorescence image of CdTe QDs under UV irradiation.


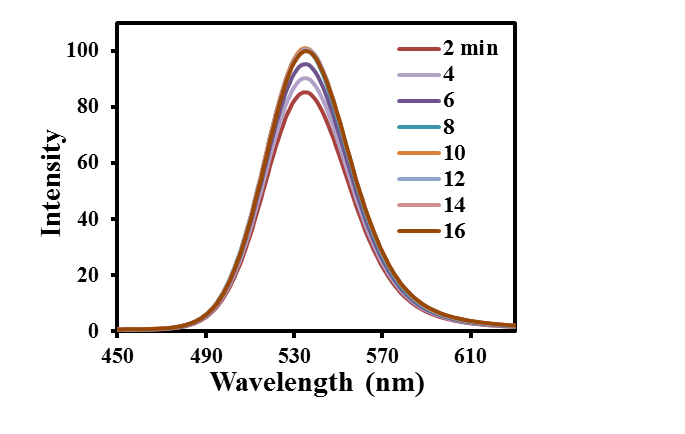
**2. Effect of NaOH on QDs’ emission**

**Figure S2.** The effect of NaOH on QDs’ emission over time (NaOH concentration was 60 mmol L^-1^ and excitation wavelength was 340 nm).

**3. The result of dynamic light scattering (DLS) measurement for QDs**

**
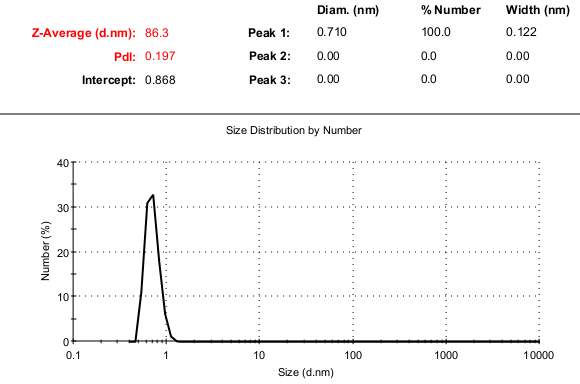
**

**Figure S3.** Number size distribution of the TGA-capped CdTe QDs.

**4. Red shift in absorption spectra of QDs in the presence of D- and L-cysteine**

**
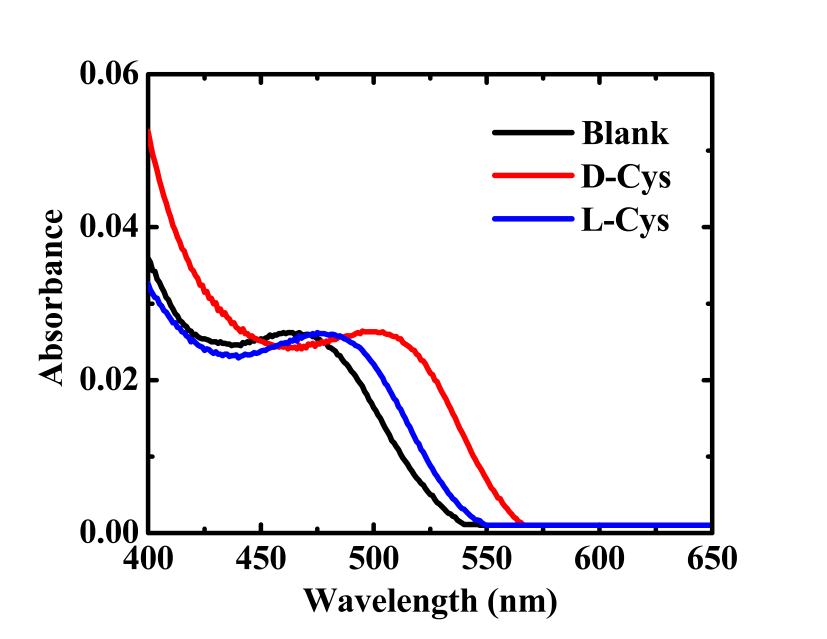
**

**Figure S4.** Absorption spectra of QDs 2 h after the addition of 2 mmol L^-1^ L- and D-cysteine (NaOH at concentration of 60 mmol L^-1^).

**5. Intensity size distribution of QDs after the addition of (a) L- and (b) D–cysteine**

**(a)**

**(b)**


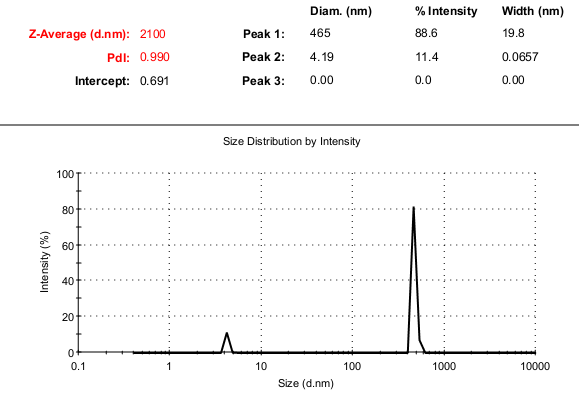

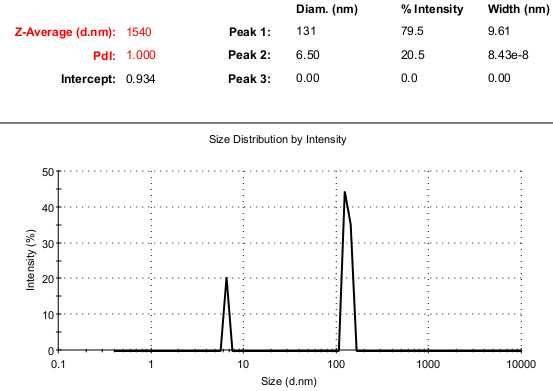


**Figure S5.** Intensity size distribution of QDs 2 h after the addition of 60 mmol L^-1^ NaOH and 2 mmol L^-1^ (a) L- and (b) D–cysteine.

**6. The effect of NaOH concentration on the difference between emission wavelengths of QDs**

**
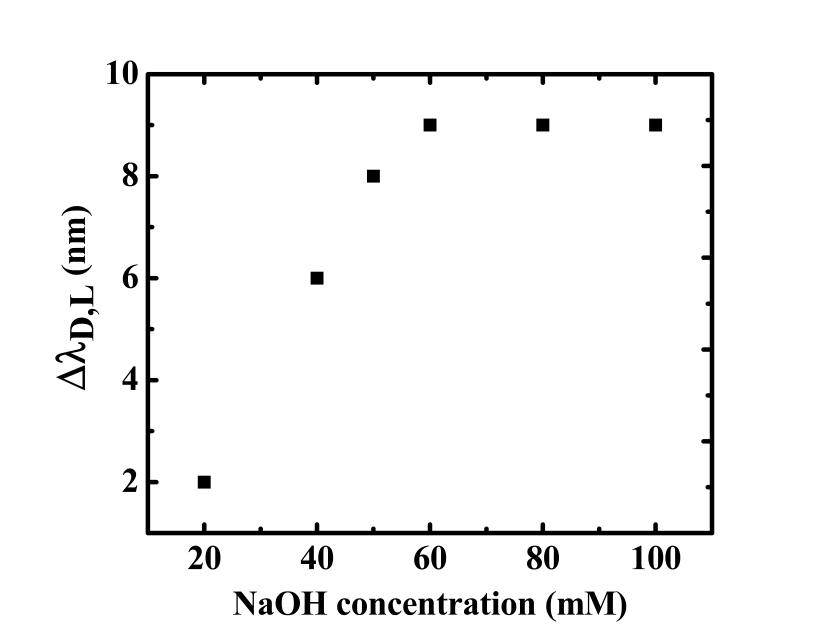
**

**Figure S6.** The effect of NaOH concentration on the difference between the maximum emission wavelengths of QDs in the presence of D- and L-cysteine, (cysteine at concentration of 1 mmol L^-1^, excitation wavelength is 340 nm, all spectra were recorded 2 h after the addition of cysteine).


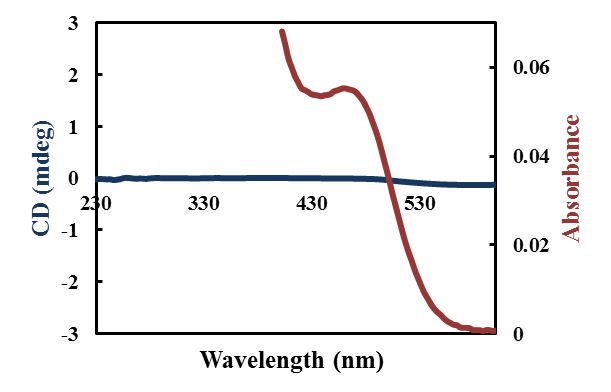
**7. CD and absorption spectra of the as-prepared CdTe QDs**

**Figure S7.** The CD (blue line) and absorption (red line) spectra of the as-prepared CdTe QDs.


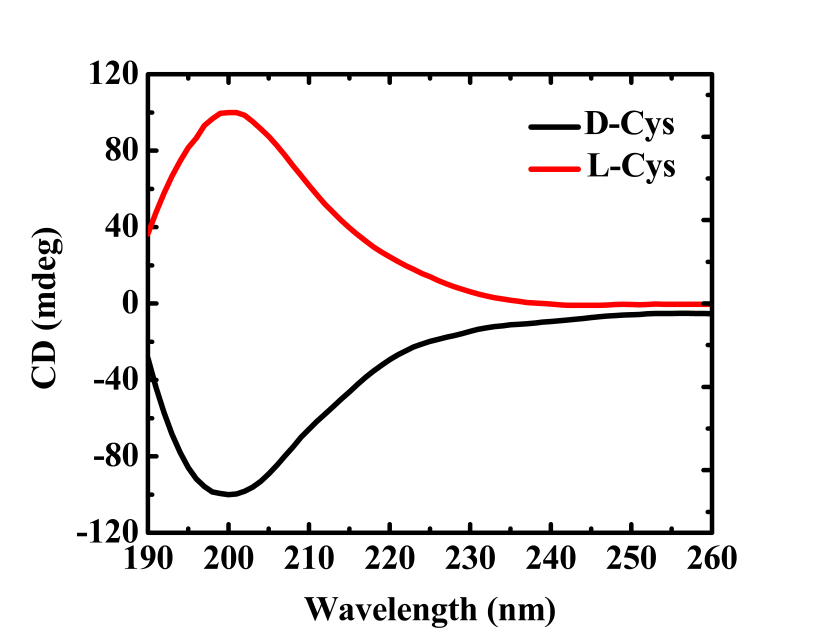
**8.** **CD spectra of L- and D-cysteine**

**Figure S8.** CD spectra of L- and D-cysteine.


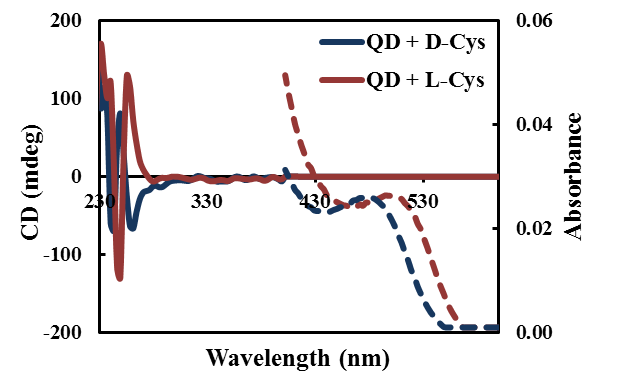
**9. CD and absorption spectra of QDs in the presence of D- and L-cysteine**

**Figure S9.** The CD (solid lines) and absorption (dotted lines) spectra of QDs 2 h after the addition of 2 mmol L^-1^ of L- and D-cysteine (NaOH at concentration of 60 mmol L^-1^).

**References**

1. Yu, W. W., Qu, L., Guo, W. & Peng, X. Experimental determination of the extinction coefficient of CdTe, CdSe, and CdS nanocrystals*. Chem. Mate*r**.** 15, 2854-2860 (2003).
